# The Departmental Method

**Published:** 1920-05-08

**Authors:** G. Q. Roberts


### THE DEPARTMENTAL METHOD.

By G. Q. Roberts, C.B.E., M.A.Oxon.

By the death of Sir Henry Burdett last week the Voluntary Hospitals of Great Britain and of London in particular have sustained the loss of an ardent supporter and a great worker in their cause. For something more than fifty years Sir Henry has been closely identified with hospitals. Having served his apprenticeship in the hospital world at the Queen's Hospital in Birmingham, he was elected Secretary of the Dreadnought Seamen's Hospital in September 1874, and for six years he devoted himself to the cause of that Hospital, bringing its work prominently before the public and laying the foundation-stone of its future greatness. In 1880 he severed his official connection with hospital life and in the following year was appointed Secretary of the Share and Loan Department of the Stock Exchange. With his work in the City it is not the intention of this article to deal. Suffice it to say that he devoted the energy and ability which were characteristic of the man to the work to which he was appointed, and he has left his mark in the great volume published yearly—"Burdett's Official Intelligence." His was the brain which conceived the outline of this work. It was he who selected those who could give him the necessary assistance in carrying out accurately the details of the Companies' Register in the Stock Exchange Department, of which he was the chief.

Neither his routine City work nor this compilation of details for which he made himself responsible satisfied his restless spirit. His whole love was for the Hospitals, and at his house, The Lodge, Porchester Square, he undertook many tasks for the direct benefit of the hospital movement of which he was throughout his life so distinguished a supporter. It was the privilege of the writer of this present article to take part in carrying out some of the details of this work. In the year 1886 he was already engaged in the compilation of the details of his book "Hospitals and Asylums of the World." With ceaseless energy he had collected in many languages from all parts of the world short descriptions of all hospitals and asylums of which he could find notice or learn any facts. He had already founded his newspaper The Hospital, and was anxiously endeavouring to secure the support and sympathy of all hospital workers, and to give them in his paper every fair opportunity of bringing before the public the work and the wants of the institutions for which they were responsible. Keenly interested in finance, he had already conceived and begun the publication of the book which is still published annually, "Burdett's Hospitals and Charities," a book which has for many years past been looked upon as the most reliable book of reference in regard to the work of the finance of hospitals. He had secured the confidence and the co-operation of all hospital managers, and from them he obtained the details as they are published yearly regarding all important facts connected with each individual hospital.

The different branches of hospital work enlisted his sympathies, but none more so than the nursing side. For many years he laboured in the nurses' cause, and as the founder of the Royal National Pension Fund for Nurses has built a monument of which any philanthropist might be proud. The energy which he threw into this movement was extraordinary. Its success was entirely due to the confidence placed in him by the heads of the great City financial houses, who subscribed so liberally to the first foundation and to the endowment fund which was the basis on which the whole fabric was built. Securing the assistance of keen workers and able actuaries, he launched his scheme, and this Fund was joined within ten years of its first foundation in 1887 by no less than 5,000 nurses, and many of these nurses are now reaping the benefit of their providence. Side by side with this Pension Fund he conceived and established the Junius S. Morgan Benevolent Fund, which has rendered so much assistance to nurses who have fallen out by the way.

It must not be imagined for one moment that any of this work was carried out without opposition. Sir Henry Burdett's character was not exactly conciliatory. His mind once fixed on a good object, he pushed ruthlessly forward to obtain his end, brushing aside his enemies, fighting their opposition, and scorning to attempt to conciliate those who opposed him. He never shrank from entering upon a controversial dispute. He took an active part in the bitter controversy in regard to the registration of nurses. In the earliest days of this movement he published a Register of Nurses, but the leaders of the nursing world, women who were devoting their lives to the betterment of the training and the improvement of the status of the trained nurse, convinced him that the time for registration had not yet come. In his weekly paper he gave the strongest encouragement to forwarding all improvements in the education and training of nurses, believing that registration should come in due time when a standard of registration acceptable to the majority had been established. The foundation of the College of Nursing, which has enlisted the sympathies of practically all the leading matrons of the hospitals and infirmaries of Great Britain, received his warmest support in this paper and in his journal, The Nursing Mirror, which has for always carried great weight with nurses.

With regard to the general conduct of hospitals Sir Henry was always a most keen advocate of the voluntary principle. From the early days, when he distinguished himself by improving the financial position of the Seamen's Hospital and practically doubled its income during his tenure of office, he believed that a hospital, to secure the support of the public, must do good work, and that it would do its best work if its existence depended on the support of the public. His pen was ever ready to

**The Departmental Method**—(*continued*).

uphold the claim of all hospitals which in his judgment deserved public sympathy. Nor did he hesitate to point out to the managers the faults which he judged to exist in institutions, the conduct of which was reviewed by him. He was a pusher, and as such he often alienated those who might have been his friends had he adopted other methods. While all must appreciate the great work he did, it is undoubtedly true that his methods of work did not always secure that support and friendship which the objects he aimed at so thoroughly deserved.

Lord Somerleyton, who has so long been connected with the great Funds of London which were founded with the object of supplementing the incomes of the various hospitals, stated at the annual meeting of the General Council of the King Edward Hospital Fund on April 30 last, "Sir Henry Burdett was in many ways the originator of the idea of King Edward's Hospital Fund." In still earlier days he had strongly supported the foundation in 1873 of the Hospital Sunday Fund, and always expressed the highest admiration for the valuable initiation of this Fund by Canon Miller. Recognising the fact that the King Edward Fund did not reach all who might wish to contribute to the hospitals and that it was administered on perhaps too large a scale to bring in small donors, he took a large part in, if he did not originate, the foundation of the League of Mercy, of which he was for many years the Honorary Treasurer.

It is difficult to conceive that any one man should wish to devote himself to so many of the branches of hospital work, but his organising power was so extraordinary that he invariably had at hand workers who were able to supply him with facts which he, in turn, was able to marshal for the edification and instruction of all persons interested in any movement in the hospital world. He was an indefatigable worker. After a busy day in the City which would have satisfied most men, he returned to "The Lodge," in Porchester Square, and after a frugal dinner and a short rest his great spirit was never satisfied unless he worked well on into the night, devoting himself, with an extraordinary grasp of detail, to any subjects which came before him either in the course of his work or in consequence of enquiries addressed to him by those who were interested in hospitals. Everyone might rest assured that on any hospital matter an enquiry addressed to Burdett was sure to receive careful consideration and a comprehensive answer. Surely such work as this should have sufficed for any man, but Burdett's character filled him with an ardent desire to right any wrong. He dealt with many political subjects, and was interested in all that concerned his fellow-men. He thought and wrote of their lives, both in health and sickness, and whilst he will be known almost entirely for his hospital work, he gave much time and original thought to political matters. He was a man of the strongest possible sympathy with all suffering. He took the most kindly interest in the misfortunes of others. He was sympathetic with all movements for the improvement of the people, but his confidence in his own judgment was such that he often failed to secure that sympathy of co-workers which his high ideals should have enlisted.

When time has softened the enmities of those who were irritated by the decision with which he invariably aimed at the end he had in view, we have no doubt that Sir Henry Burdett will live long in the memory of all for the great work in which he was so distinguished a leader.

